# Correlates of COVID-19 Vaccine Uptake in Black Adults Residing in Allegheny County, PA

**DOI:** 10.1089/heq.2022.0215

**Published:** 2023-08-23

**Authors:** Ashley V. Hill, Harika P. Dyer, John Gianakas, Ruth Howze, Ayanna King, Tiffany L. Gary-Webb, Dara D. Méndez

**Affiliations:** ^1^ Department of Epidemiology, University of Pittsburgh School of Public Health, Pittsburgh, Pennsylvania, USA.; ^2^ Black Equity Coalition, Pittsburgh, Pennsylvania, USA.; ^3^ Epidemiology Data Center, University of Pittsburgh, Pittsburgh, Pennsylvania, USA.

**Keywords:** COVID-19, vaccination, vaccine hesitancy, black adults

## Abstract

**Introduction:**

Efforts to address vaccine uptake and access among black adults will be relevant for continued coronavirus disease 2019 (COVID-19) eradication efforts and can be transferable to other prevention efforts in future pandemics. This study investigated factors related to COVID-19 vaccine uptake and access among black residents in Allegheny County, PA.

**Methods:**

Surveys were administered electronically from October 2021 to January 2022 to black Allegheny County residents aged 18 and older. Questions included thoughts on COVID mitigation strategies (e.g., masking, social distancing), vaccination status, intention to vaccinate children, trust of COVID-19 information sources and vaccines, family needs, access to support services, and social media use to access information. Descriptive statistics and significant correlates of being vaccinated using adjusted logistic regression models are reported.

**Results:**

Of the overall sample (*N*=397), the majority were fully vaccinated (*n*=306, 77%). Fully vaccinated participants were more likely to be female (62.5%, *p*=0.010), age 60 years or older (34.3%, *p*=0.0002), have some college education (23.2%, *p*<0.0001), and be employed full time (50.0%, *p*=0.0001) compared with nonvaccinated individuals. Among the unvaccinated participants (*n*=91), the primary reason was fear of illness (8.9%), long-term effects (6.5%), mistrust in the vaccine (6.3%), and needing more information (4.5%). Vaccine-hesitant participants were more likely to be unvaccinated (adjusted odds ratio=2.3, 95% confidence interval 1.25–4.14) after adjusting for age, education, employment, insurance, health status, and income.

**Conclusion:**

Vaccine hesitancy may be improved by directly addressing fear of illness resulting from vaccines and improving clarity in the vaccine development and approval process to improve uptake among black adults.

## Introduction

Coronavirus disease 2019 (COVID-19) has become a leading cause of death in the United States with devastating social and economic consequences.^1^ In December 2020, the Pfizer-BioNTech COVID-19 vaccine was approved for emergency authorization use by the Food and Drug Administration (FDA).^2^ In clinical trials and vaccine effectiveness studies, all the FDA-approved vaccines have demonstrated the efficacy to protect against severe COVID-19 infections, prevent mortality, and reduce the spread of disease.^2^ During the emergence of the Omicron COVID-19 variant between December 2021 and January 2022, unvaccinated individuals were at significantly greater risk of COVID-19 infection (incidence rate ratio=3.1, 95% confidence interval [CI] 1.7–5.8) compared with people vaccinated with a completed primary series of Pfizer-BioNTech, Moderna mRNA, or Janssen vaccines.^3^

Unvaccinated adults were hospitalized at 12 times the rate of vaccinated adults who received a primary series with a booster or additional dose and 4 times the rate of vaccinated adults who received the primary series but no booster.^4^ People aged 12 and older who were fully vaccinated with a booster had a 14.9 times decreased risk of dying from COVID-19 in September 2022.^5^ In total, COVID-19 vaccination has prevented an estimated 14.4 million deaths globally in 2021.^6^

Although black Americans are disproportionately more likely to experience infection, hospitalization, and death from COVID-19 compared with non-Hispanic white Americans, disparities in vaccination continue to persist in black Americans.^7,8^ In May 2021, 25% of black adults had been vaccinated, compared with 27% of Hispanics, 39% of whites, and 48% of Asians in the United States.^9^ In Allegheny County, Pennsylvania, disparities in COVID-19 vaccination rates followed similar national patterns. In March 2021, black residents were 8.6% of vaccine recipients, but 12.8% of the population, compared with white residents who were 86.2% of vaccine recipients and 78% of the population.^10^ Although vaccine uptake among black residents in Allegheny County has improved, as of October 2022, vaccine rates are still lower in black Allegheny County residents (54% black, 68.9% white).^11^

Vaccine hesitancy has been publicly suggested as the major reason for insufficient uptake in black communities for a myriad of reasons, including medical mistrust, structural racism, historic government-sanctioned ethical atrocities, and misunderstanding of vaccinology.^12^ In addition, vaccine distribution across Pennsylvania has not taken an equitable approach to center communities disproportionately affected by COVID-19. Identifying actual versus perceived challenges to vaccine uptake is vital to improving public health efforts to mitigate the pandemic.^13^

Vaccine hesitancy is defined by the Vaccine Hesitancy Framework of the World Health Organization's Strategic Advisory Group of Experts on Immunization (SAGE Working Group) as “a delay in acceptance or refusal of vaccination despite availability of vaccination services.”^14^ The SAGE Working Group's framework suggests that influencing factors for vaccine uptake are confidence (trust in vaccines and the systems that deliver them), complacency (perceived disease risk and the importance of the vaccine), and convenience (physical availability, affordability, service quality, and accessibility).

This framework, while useful for approaching vaccine distribution, places emphasis on individual decision-making but neglects to incorporate perspectives on structural barriers to vaccination acceptance and historical disinvestment in excluded and oppressed communities such as black communities.^15,16^ For example, institutional distrust of medicine and health care,^17–21^ particularly within black American communities, is rooted in historical unethical experimentation and continued medical racism, well-documented influencers of medical mistrust.^22,23^

Similarly, questions surrounding governmental transparency, politicization of public health, and concerns of vaccine safety contribute to vaccine hesitancy in the United States.^24–27^ Vaccine hesitancy is a multifaceted issue that represents a potentially critical barrier to effective vaccination and the subsequent improved health outcomes for a myriad of issues. As such, it is important to understand the multiple factors that influence COVID-19 vaccination decision-making among black Americans.

Allegheny County, Pennsylvania, is a prime location to examine factors related to vaccine uptake and hesitancy. Allegheny County has undergone several evaluations suggesting that social inequities and disparities are stark and that the need for approaches that investigate and remedy these challenges is immediately needed.^28^ Investigations into vaccine intentions have already been undertaken in the county, particularly among parents of young children.^29–31^ Considering the persistent health inequities and anticipated inequities in COVID-19, a group of concerned colleagues came together in April 2020 to discuss relevant issues and push for the consistent collection and reporting of COVID-19 data by race and ethnicity in Allegheny County to better understand and address the impact on black communities in the region.^13^ The Black Equity Coalition (BEC) was formed (blackequitypgh.org) to support the creation of equitable systems to affirm the dignity of every human being through collaborations, networks, and policymaking.

Inequities in vaccine distribution and understandings of reduced uptake in marginalized communities will preclude the elimination of COVID-19 and must quickly be addressed to end the pandemic. The BEC recognized that equitable distribution of vaccines requires leveraging existing relationships among the local network of advocates, scientists, physicians, funders, and community members engaged in COVID pandemic response and equity-related efforts. To enhance the understanding of influencing factors of COVID-19 vaccine uptake among black adults locally, the purpose of this study was to examine correlates of vaccine uptake in black adults in Allegheny County, Pennsylvania, and to describe the prevalence and rationale for vaccine hesitancy among the sample.

## Methods

### Procedures and population

Electronic surveys were collected in person at community events and online from September 1, 2021, to January 10, 2022. Research staff were available in person to guide any participant who desired assistance completing surveys. Participants were eligible if they self-identified as black or African American, were at least 18 years of age, and were current residents of Allegheny County, Pennsylvania. Recruitment came from community and vaccination events coordinated through the BEC and were conducted in neighborhoods identified to be priorities for improving equity by the Racial and Ethnic Approaches to Community Health (REACH) program,^32^ a program supporting the understanding and implementation of health equity strategies for black communities in Allegheny County.^33^

For online surveys, a public survey link was disseminated via physical flyers posted throughout community centers, via email and online through community-based organization collaborators, as well as social media. Surveys were completed through Qualtrics, an internet-based survey system, either using the participant's own device or a study-provided tablet at in-person community events. Written informed consent was obtained before survey completion, identifying information was only obtained to provide incentive payment, was not linked to survey responses, and all participants received an incentive for completion.

### Measures

The study team assembled a survey of ∼95 questions examining the following domains: participant demographic characteristics; current or intended vaccination status and perspectives on COVID-19 vaccine safety; intention to vaccinate children; desired locations of vaccine clinics; general trust or mistrust of COVID-19 information and vaccines; health care access and resource information; individual needs and access to resources; social media use; and smoking behaviors (Supplementary Appendix SA1).

### Demographic characteristics

A subset of the NIH RADx Executive Committee-Required Common Data Elements (CDEs) for studies of COVID-19^34^ were included. Participants were asked to report their ages: 18–30, 31–40, 41–50, 51–60, and >60; education: less than high school degree, high school degree, some college/college alternative, bachelor's degree or greater; employment: employed full time, employed part-time, retired, other (e.g., student and full-time or part-time employment); income: <$20,000, $20,000–$49,999, >$50,000; and insurance status: private (i.e., employee sponsored), Medicaid or other government insurance, Medicare, or none. Self-reported health was assessed as excellent/very good, good, fair/poor. The purpose of truncating participants' ages was to more closely examine younger individuals in the sample. This was desired to understand more about the younger people and their perspectives on COVID-19 and as less is known about their reasons for being unvaccinated in the literature.

In addition, participants were asked if they had a primary care physician (yes/no), if they had ever tested positive for COVID-19 (yes/no), and if they were parents, whether their children had been or would be vaccinated against COVID-19 (yes/no). Additional questions about infection mitigation behaviors were also included, such as masking practices or the ability to work from home. Participants were also asked where they obtained their information related to COVID-19 infection, preventing infection, vaccines, and where they seek medical care if needed.

### Primary outcome

The primary outcome for this study was being unvaccinated. Participants were asked “Are you currently fully vaccinated for COVID-19 (meaning you received 1 shot of Johnson & Johnson's Janssen or 2 shots of Moderna or Pfizer-BioNTech)?” Answer choices were “yes” or “no.” Follow-up questions were asked to determine whether those who responded “no” were partially vaccinated or fully unvaccinated, and whether they intended to become fully vaccinated.

### Primary predictor—Hesitancy Scale

At the time of this study, there was only one published scale examining COVID-19 vaccine hesitancy and medical mistrust among black adults in the United States. An adaptation of this scale was included as the primary predictor of vaccination among the Allegheny County sample; internal reliability and validity were confirmed after collection (Cronbach's *α*: 0.86). Questions asked general agreement or disagreement with statements such as “A lot of information about COVID-19 is being held back by the government” and “There is a cure for COVID-19, but it is being withheld from black people.” Responses ranged from strongly agree^1^ to strongly disagree^5^ and positively worded prompts were reverse coded so that higher scores indicated greater mistrust.

A mean summary score was created for each respondent and the overall sample median hesitancy score was calculated. Each respondent was dichotomized based on the sample median (median=3.00); individuals whose vaccine hesitancy score fell below the sample median were characterized as “not vaccine hesitant,” and individuals whose vaccine hesitancy score fell above the sample median were characterized as “vaccine hesitant.”

### Statistical analysis

Frequencies (*n*) and percentages (%) are reported for the total sample and stratified by vaccination status. Chi-square tests for homogeneity between vaccinated and unvaccinated samples were conducted. Logistic regression was used to model the probability of being unvaccinated. Regression models were adjusted for age, education, employment, insurance, health status, and income. Unadjusted and adjusted odds ratios (aORs) are reported with 95% CIs (SAS V9.4).

## Results

A total of 397 participants were included in the study (Table 1). The majority of participants were women (62.5%), and almost 30% of the sample was older than 60 years. Roughly half (48.9%) of the sample completed some college or a college alternative, reported working full time (47.1%), and an annual income between $20,000 and $49,999 (47.3%). Nearly a third of the sample (32.3%) had private insurance, and over three-quarters (79.8%) had a primary care physician. A small proportion (13.4%) of participants had previously tested positive for COVID-19; a majority (77.1%) of the sample reported being fully vaccinated against COVID-19. Chi-square tests indicated significant differences in characteristics between vaccinated and unvaccinated participants.

**Table 1.** Sample Characteristics, Including Demographics, Self-Reported Coronavirus Disease 2019 Positivity, and Parent Intention to Vaccinate Children, by Vaccination Status, Allegheny County, PA, September–December 2021

	Total (***N***=397)	Are you currently fully vaccinated for COVID-19 (received 1 shot of J3 or 2 shots of Moderna or Pfizer)?		***p***
		**Yes (*n*=306, 77.1%)**	**No (*n*=91, 22.9%)**	
Gender, *n* (%)				0.01^a^
Male	123 (31.0)	105 (34.3)	18 (19.8)	
Female	248 (62.5)	185 (60.5)	63 (69.2)	
Other (intersex, none of these describe me, prefer not to answer)	26 (6.5)	16 (5.20)	10 (11.0)	
Age groups, *n* (%)				<0.01^a^
18–30	78 (19.6)	48 (15.7)	30 (33.0)	
31–40	85 (21.4)	64 (20.9)	21 (23.1)	
41–50	53 (13.4)	42 (13.7)	11 (12.1)	
51–60	64 (16.1)	47 (15.4)	17 (18.7)	
60+	117 (29.5)	105 (34.3)	12 (13.2)	
Education, *n* (%)				<0.01^a^
Less than high school	15 (3.80)	8 (2.60)	7 (7.90)	
High school degree	77 (19.6)	49 (16.1)	28 (31.5)	
Some college or college alternative	192 (48.9)	144 (47.4)	48 (53.9)	
Bachelor's degree or greater	109 (27.7)	103 (33.9)	6 (6.70)	
Employment, *n* (%)				<0.01^a^
Full time	185 (47.1)	152 (50.0)	33 (37.1)	
Part-time	61 (15.5)	39 (12.8)	22 (24.7)	
Retired	64 (16.3)	58 (19.1)	6 (6.70)	
Other (unemployed, student)	83 (21.1)	55 (18.1)	28 (31.5)	
Income, *n* (%)				<0.01^a^
Less than $20,000	130 (32.9)	83 (27.2)	47 (52.2)	
$20,000 to $49,999	187 (47.3)	151 (49.5)	36 (40.0)	
$50,000 or more	78 (19.7)	71 (23.3)	7 (7.80)	
Insurance, *n* (%)				<0.01^a^
Private	127 (32.3)	119 (39.3)	8 (8.90)	
Medicaid and other government	143 (36.4)	96 (31.7)	47 (52.2)	
Medicare	97 (24.7)	78 (25.7)	19 (21.1)	
None	26 (6.60)	10 (3.30)	16 (17.8)	
General health, *n* (%)				0.01^a^
Excellent/very good	163 (41.3)	130 (42.6)	33 (36.7)	
Good	154 (39.0)	125 (41.0)	29 (32.2)	
Fair/poor	78 (19.7)	50 (16.4)	28 (31.1)	
Do you currently have a primary care physician? *n* (%)				<0.01^a^
No	80 (20.2)	48 (15.7)	32 (35.2)	
Hesitancy score predictor (classified via sample median), *n* (%)				<0.01^b^
Hesitant	112 (36.6)	62 (68.1)	174 (43.8)	
Have you tested positive for covid?				0.0885^a^
Yes	53 (13.4)	36 (11.8)	17 (18.7)	
No	344 (86.6)	270 (88.2)	74 (81.3)	
Has your child(ren) (aged 12 and older) received the vaccine for COVID-19? *n* (%)				<0.01^a^
N/A: have no children	205 (51.6)	170 (55.6)	35 (38.5)	
N/A: No children 12 or older	113 (28.5)	77 (25.2)	36 (39.6)	
Yes	45 (11.3)	41 (13.4)	4 (4.40)	
No	34 (8.60)	18 (5.90)	16 (17.6)	
Do you plan to have your child(ren) (younger than 12) receive the vaccine for COVID-19 when it is approved? *n* (%)				<0.01^a^
N/A: have no children	205 (51.6)	170 (55.6)	35 (38.5)	
N/A: No children younger than 12	50 (12.6)	38 (12.4)	12 (13.2)	
Yes	72 (18.1)	63 (20.6)	9 (9.90)	
No	37 (9.30)	16 (5.20)	21 (23.1)	
Not sure/maybe	33 (8.30)	19 (6.20)	14 (15.4)	

^a^ Chi-square *p*-value.

^b^ Kruskal–Wallis *p*-value.

COVID-19, coronavirus disease 2019.

Vaccinated participants were more likely to be older (*p*<0.01), employed full time (*p*<0.01), report greater income (*p*<0.01), have private insurance (*p*<0.01), report better health (*p*<0.01), and have a primary care physician (*p*<0.01). A smaller proportion of vaccinated participants were categorized as COVID-19 vaccine hesitant (37.9%) compared with unvaccinated participants (69.2%, *p*<0.01).

Participants were asked 12 questions assessing their precautionary and protective behaviors against COVID-19 (Table 2). Social distancing (80.1%), mask wearing (82.9%), and seeking out information about COVID-19 on social media (54.8%) were primarily endorsed among respondents. Some participants said that they were able to maintain a social bubble (32.0%), have groceries delivered (19.4%), gather outdoors (24.7%), pick up food to eat at home (40.3%), work remotely (30.5%), and participate in virtual gatherings (43.3%). Vaccinated adults were significantly more likely to participate in large gathering outdoors (28.1% vs. 13.2%, *p*<0.01) and work remotely (34.6% vs. 16.5%, *p*<0.01). When asked about their frequency of mask usage, a greater proportion of vaccinated participants reported wearing masks “all of the time” or “most of the time” (81.0%) compared with unvaccinated participants (63.8%).

**Table 2.** Participant Self-Reported Coronavirus Disease 2019 Safety Precautions and Practices by Vaccination Status

	Total (***N***=397)	Are you currently fully vaccinated for COVID-19 (received 1 shot of J3 or 2 shots of Moderna or Pfizer)?		***p*** ^a^
		**Yes (*n*=306, 77.1%)**	**No (*n*=91, 22.9%)**	
Social distancing, *n* (%)				0.57
No	79 (19.9)	59 (19.3)	20 (22.0)	
Maintained a social bubble, *n* (%)				0.43
No	270 (68.0)	205 (67.0)	65 (71.4)	
Wore a mask, *n* (%)				0.28
No	68 (17.1)	49 (16.0)	19 (20.9)	
How often do you wear a mask? *n* (%)				<0.01
(1) All the time	176 (44.3)	142 (46.4)	34 (37.4)	
(2) Most of the time	130 (32.7)	106 (34.6)	24 (26.4)	
(3) Often	51 (12.8)	37 (12.1)	14 (15.4)	
(4) Sometimes	32 (8.10)	18 (5.90)	14 (15.4)	
(5) Rarely	7 (1.80)	2 (0.70)	5 (5.50)	
(6) Never	1 (0.30)	1 (0.30)	0 (0.00)	
Delivered food or groceries, *n* (%)				0.09
No	320 (80.6)	241 (78.8)	79 (86.8)	
Gathered outdoors, *n* (%)				<0.01
No	299 (75.3)	220 (71.9)	79 (86.8)	
Picked up food and ate at home, *n* (%)				0.26
No	237 (59.7)	178 (58.2)	59 (64.8)	
Worked remotely, *n* (%)				<0.01
No	276 (69.5)	200 (65.4)	76 (83.5)	
Participated in virtual meetings, ceremonies, religious celebrations, and/or gatherings, *n* (%)				0.07
No	225 (56.7)	166 (54.2)	59 (64.8)	
COVID safety measures: other, *n* (%)				0.88
No	392 (98.7)	302 (98.7)	90 (98.9)	
Have you sought out information on COVID-19 vaccination on social media? *n* (%)				0.42
Yes	178 (45.2)	134 (44.1)	44 (48.9)	
No	216 (54.8)	170 (55.9)	46 (51.1)	

^a^ Chi-square *p*-value.

Vaccine hesitancy was reported in more than half the sample (54.9%; mean=3.06, standard deviation: 0.76; median=3.00). Participants primarily chose to neither agree nor disagree to a majority of statements. These included items such as “A lot of information about COVID-19 is being held back by the government,” “COVID-19 is man-made,” “When it comes to COVID-19, black people will receive the same medical care from health care providers as other groups,” and “When it comes to COVID-19, black people cannot trust health care providers” (Table 3). In addition, unvaccinated participants were asked to report reasons they chose not to get vaccinated. Fear of illness (39.8%), not knowing the long-term effects of the vaccine (33.0%), and fear of experiencing side effects (26.4%) were the most commonly reported reasons for being unvaccinated (Table 4).

**Table 3.** Participant Responses to Investigator-Adapted Vaccine Hesitancy Questionnaire

Vaccine Hesitancy Scale, Cronbach's alpha	0.86
Mean (SD)	3.08 (0.75)
Median (range)	3.00 (1.00–4.82)
	Total (*N*=397)

A lot of information about COVID-19 is being held back by the government, *n* (%)	
(1) Strongly agree	89 (22.4)
(2) Agree	106 (26.7)
(3) Neither agree nor disagree	126 (31.7)
(4) Disagree	63 (15.9)
(5) Strongly disagree	13 (3.30)
The government cannot be trusted to tell the truth about COVID-19, *n* (%)	
(1) Strongly agree	66 (16.8)
(2) Agree	98 (24.9)
(3) Neither agree nor disagree	125 (31.8)
(4) Disagree	83 (21.1)
(5) Strongly disagree	21 (5.30)
The government is hiding information about COVID-19, *n* (%)	
(1) Strongly agree	64 (16.2)
(2) Agree	104 (26.4)
(3) Neither agree nor disagree	127 (32.2)
(4) Disagree	77 (19.5)
(5) Strongly disagree	22 (5.60)
Black people should be suspicious of information from the government about COVID-19, *n* (%)	
(1) Strongly agree	57 (14.4)
(2) Agree	99 (25.1)
(3) Neither agree nor disagree	107 (27.1)
(4) Disagree	95 (24.1)
(5) Strongly disagree	37 (9.40)
When it comes to COVID-19, the government is lying to us, *n* (%)	
(1) Strongly agree	40 (10.2)
(2) Agree	83 (21.1)
(3) Neither agree nor disagree	139 (35.3)
(4) Disagree	93 (23.6)
(5) Strongly disagree	39 (9.90)
COVID-19 is man-made, *n* (%)	
(1) Strongly agree	60 (15.4)
(2) Agree	84 (21.6)
(3) Neither agree nor disagree	137 (35.2)
(4) Disagree	74 (19.0)
(5) Strongly disagree	34 (8.70)
There is a cure for COVID-19, but it is being withheld from black people, *n* (%)	
(1) Strongly agree	21 (5.30)
(2) Agree	42 (10.7)
(3) Neither agree nor disagree	132 (33.5)
(4) Disagree	131 (33.2)
(5) Strongly disagree	68 (17.3)
When it comes to COVID-19, black people cannot trust health care providers, *n* (%)	
(1) Strongly agree	33 (8.40)
(2) Agree	57 (14.5)
(3) Neither agree nor disagree	137 (34.8)
(4) Disagree	120 (30.5)
(5) Strongly disagree	47 (11.9)
When it comes to COVID-19, doctors have the best interests of patients in mind,^a^ *n* (%)	
(1) Strongly disagree	9 (2.30)
(2) Disagree	37 (9.30)
(3) Neither agree nor disagree	128 (32.3)
(4) Agree	168 (42.4)
(5) Strongly agree	54 (13.6)
When it comes to COVID-19, black people will receive the same medical care from health care providers as other groups,^a^ *n* (%)	
(1) Strongly disagree	43 (10.9)
(2) Disagree	93 (23.6)
(3) Neither agree nor disagree	117 (29.7)
(4) Agree	103 (26.1)
(5) Strongly agree	38 (9.60)
I have more trust in the Biden administration compared with the Trump administration when it comes to controlling the spread and treatment of COVID,^a^ *n* (%)	
(1) Strongly disagree	14 (3.60)
(2) Disagree	27 (6.90)
(3) Neither agree nor disagree	85 (21.6)
(4) Agree	113 (28.7)
(5) Strongly agree	155 (39.3)

Adapted from: Bogart et al.^26^

^a^ Indicates responses were reverse coded to generate mean summary score.

SD, standard deviation.

**Table 4.** Participant-Reported Reasons for Vaccine Hesitancy Among Unvaccinated Participants by Age Category

	Age						***p*** ^a^
	**18–30 (*N*=30)**	**31–40 (*N*=21)**	**41–50 (*N*=11)**	**51–60 (*N*=17)**	**60+ (*N*=12)**	**Total**	
						**(*N*=91)**	
Reason you do not intend to get the vaccine or may not get the vaccine, *n* (%)							0.13171
N/A: plans on getting vaccine	2 (6.70)	2 (10.0)	3 (30.0)	4 (25.0)	4 (33.3)	15 (17.0)	
Does not trust	9 (30.0)	5 (25.0)	1 (10.0)	8 (50.0)	2 (16.7)	25 (28.4)	
Fear of illness	12 (40.0)	9 (45.0)	5 (50.0)	3 (18.8)	6 (50.0)	35 (39.8)	
Does not trust and fear of illness	7 (23.3)	4 (20.0)	1 (10.0)	1 (6.30)	0 (0.00)	13 (14.8)	
Missing	0	1	1	1	0	3	
I'm waiting to see, *n* (%)							0.05541
N/A: plans on getting vaccine	2 (6.70)	2 (9.50)	3 (27.3)	4 (23.5)	4 (33.3)	15 (16.5)	
No	19 (63.3)	17 (81.0)	8 (72.7)	9 (52.9)	8 (66.7)	61 (67.0)	
Yes	9 (30.0)	2 (9.50)	0 (0.00)	4 (23.5)	0 (0.00)	15 (16.5)	
I need more information, *n* (%)							0.29091
N/A: plans on getting vaccine	2 (6.70)	2 (9.50)	3 (27.3)	4 (23.5)	4 (33.3)	15 (16.5)	
No	17 (56.7)	14 (66.7)	6 (54.5)	10 (58.8)	7 (58.3)	54 (59.3)	
Yes	11 (36.7)	5 (23.8)	2 (18.2)	3 (17.6)	1 (8.30)	22 (24.2)	
I'm scared to get sick of experiencing side effects, *n* (%)							0.21911
N/A: plans on getting vaccine	2 (6.70)	2 (9.50)	3 (27.3)	4 (23.5)	4 (33.3)	15 (16.5)	
No	17 (56.7)	12 (57.1)	7 (63.6)	11 (64.7)	5 (41.7)	52 (57.1)	
Yes	11 (36.7)	7 (33.3)	1 (9.10)	2 (11.8)	3 (25.0)	24 (26.4)	
I don't know the long-term effects, *n* (%)							0.13241
N/A: plans on getting vaccine	2 (6.70)	2 (9.50)	3 (27.3)	4 (23.5)	4 (33.3)	15 (16.5)	
No	15 (50.0)	9 (42.9)	5 (45.5)	11 (64.7)	6 (50.0)	46 (50.5)	
Yes	13 (43.3)	10 (47.6)	3 (27.3)	2 (11.8)	2 (16.7)	30 (33.0)	
I think the vaccine could give me COVID, *n* (%)							0.19251
N/A: plans on getting vaccine	2 (6.70)	2 (9.50)	3 (27.3)	4 (23.5)	4 (33.3)	15 (16.5)	
No	25 (83.3)	16 (76.2)	6 (54.5)	13 (76.5)	8 (66.7)	68 (74.7)	
Yes	3 (10.0)	3 (14.3)	2 (18.2)	0 (0.00)	0 (0.00)	8 (8.80)	
Did not or did not intend to get vaccine: other reasons, *n* (%)							0.11781
N/A: plans on getting vaccine	2 (6.70)	2 (9.50)	3 (27.3)	4 (23.5)	4 (33.3)	15 (16.5)	
No	24 (80.0)	15 (71.4)	7 (63.6)	8 (47.1)	4 (33.3)	58 (63.7)	
Yes	4 (13.3)	4 (19.0)	1 (9.10)	5 (29.4)	4 (33.3)	18 (19.8)	

^a^ Chi-square *p*-value.

Roughly 17% (*n*=15) of participants described the intention to be vaccinated but had not received a dose at the time of the survey.

The odds of being unvaccinated were examined across several participant characteristics, examining overall vaccine hesitancy (Table 5). Participants who were vaccine hesitant had significantly greater odds of being unvaccinated (odds ratio [OR]=3.7, 95% CI 1.30–10.2) compared with respondents who were considered not hesitant. Effects remained significant (aOR=2.3, 95% CI 1.25–4.14) after adjusting for age, education, employment, insurance, health status, and income. Younger age (18–30; aOR=3.7–95%, CI 1.304–10.267), lower levels of education (less than high school: aOR=5.87, 95% CI 1.27–27.1; high school graduate: aOR=4.873, 95% CI 1.56–15.2; some college: aOR=3.23, 95% CI 1.15–9.13), being a Medicaid recipient (aOR=2.66, 95% CI 1.06–6.66), or having no insurance (aOR=12.0, 95% CI 3.67–39.5) was significantly associated with being unvaccinated in the sample.

**Table 5.** Results of Logistic Regression Modeling the Probability of Being Unvaccinated Among Participants Classified as Hesitant Versus Not Hesitant

	Unadjusted model	Fully adjusted model
	OR (95% CI)	aOR (95% CI)^a^
Hesitancy score		
Not hesitant (≥3.0 [sample median])	Ref	Ref
Hesitant (<3.0)	3.69 (2.23–6.09)^*^	2.27 (1.25–4.14)^*^
Age groups		
18–30	5.47 (2.58–11.6)^*^	3.66 (1.30–10.4)^*^
31–40	2.87 (1.32–6.23)^*^	2.40 (0.83–6.90)
41–50	2.29 (0.938–5.60)	1.84 (0.57–5.97)
51–60	3.16 (1.40–7.15)^*^	2.15 (0.75–6.20)
60+	Ref	Ref
Education		
Less than high school	15.02 (4.07–55.5)^*^	5.87 (1.27–27.1)^*^
High school degree	9.81 (3.81–25.2)^*^	4.87 (1.56–15.2)^*^
Some college or college alternative	5.72 (2.36–13.9)^*^	3.238 (1.15–9.13)^*^
Bachelor's degree or greater	Ref	Ref
Employment		
Full time	Ref	Ref
Part-time	2.60 (1.37–4.95)^*^	1.39 (0.60–3.19)
Retired	0.48 (0.19–1.20)	1.00 (0.28–3.60)
Other	2.35 (1.30–4.23)^*^	1.375 (0.61–3.13)
Income		
Less than $20,000	5.74 (2.44–13.5)^*^	0.88 (0.29–2.67)
$20,000 to $49,999	2.42 (1.03–5.70)^*^	1.13 (0.37–3.96)
$50,000 or more	Ref	Ref
Insurance		
Private	Ref	Ref
Medicaid and other government	7.28 (3.28–16.1)^*^	2.66 (1.06–6.66)^*^
Medicare	3.62 (1.51–8.68)^*^	1.92 (0.71–5.22)
None	23.80 (8.20–69.1)^*^	12.04 (3.67–39.6)^*^
General health		
Excellent/very good	Ref	Ref
Good	0.91 (0.52–1.59)	0.874 (0.43–1.76)
Fair/poor	2.21 (1.21–4.02)^*^	1.364 (0.63–2.95)

^a^ Adjusted for: age, education, employment, insurance, health status, and income.

^*^ Statistical significance (*p*<0.05).

aOR, adjusted odds ratio; OR, odds ratio.

## Discussion

In this sample, participants had a higher vaccination rate than black adults overall in Allegheny County (77.1% vs. 54% vaccinated).^35^ Women were overrepresented in the study population compared with county population estimates (62.5% vs. 51%)^36^ and reported similar vaccination rates (76.4% vaccinated) to the county (71.7% vaccinated).^35^ Men in the study population reported higher vaccination rates (85.4% vaccinated) than county estimates (65.7%). Study participants were primarily older than 60 years, had some college education, were working full time, and reported moderate income. Participants primarily reported engaging in protective behaviors such as wearing a mask and social distancing when they could, which is consistent with previous evaluations of adherence to COVID-19 mitigation strategies among black adults.^37,38^

Age, education, and insurance were significantly associated with vaccine hesitancy in the study adjusted models. Recent literature suggests similar findings, with age,^39–41^ education,^27,40,42^ insurance type,^43,44^ and income being associated with vaccine hesitancy. For instance, a survey of COVID-19 vaccine uptake in adults in Arkansas from October 2020 to January 2021 found that vaccine hesitancy increased with age, lower education, and among black versus white respondents.^40^

Older adults are more likely to get very sick from COVID-19, which increases risk of hospitalization, intensive care, ventilators, and death.^45^ Media attention has primarily focused on impacts of COVID-19 among older populations, and emphasis in vaccine rollout has continuously prioritized older adults, potentially leading to younger individuals perceiving a reduced risk of infection and impact on quality of life after infection.^46,47^ This reduction in perceived risk may be aligned with younger adults' perception and confidence in their own health and immunity, in turn, influencing their vaccine decisions.^48^

The most common reason for being unvaccinated in the sample was not knowing the long-term effects of the vaccine. While the prevalence of unvaccinated status was low, among those unvaccinated, roughly 63.8% reported wearing a face mask often or all the time, and 71% reported wearing a face mask the same amount or more often than earlier in the pandemic, suggesting perceived awareness of risk. There is evidence to suggest that unvaccinated individuals are aware of their risk but have not had their vaccine concerns addressed.^49^ Understanding and addressing reasons for hesitancy may be helpful among concerned individuals who are potentially interested in averting infection but have hesitations about other factors related to vaccines, such as long-term safety and transparency around development and approval. Vaccinated adults in this study expressed a desire to prevent infection as their primary reason to be vaccinated.

This may also be the case among unvaccinated adults, although they are typically not asked their concern for health and safety aside from issues with vaccine uptake. It is plausible that all adults, specifically black adults, may have concerns about vaccine before getting them and better understanding of the variability around vaccine issues would allow for greater targeted health communication in the further distribution of vaccines.

Vaccine hesitancy was prevalent and related to mistrust of government and leadership, but did not prevent black adults from being vaccinated in this study. Roughly half (49.1%) of participants agreed or strongly agreed that a lot of information about COVID-19 is being held back by the government. This was also found in a separate sample of black adults living with HIV,^26^ where participants reporting feelings that the US government was withholding information about COVID-19, which was related to their mistrust in the vaccine. Combating misinformation may be one way to increase vaccination among hesitant individuals. Specifically, information related to clarity and transparency through the development, safety, and efficacy of vaccines could be helpful.

Community-based participatory research frameworks often champion trusted community messengers to deliver vital health information in settings where medical trust is limited or where clarity and transparency related to the health outcome are reported. For instance, investigators from the Centers for Disease Control and Prevention (CDC) used a Community-Based Participatory Research framework and championed black leaders in faith-based communities to conduct a community needs assessment, and distribute 230 messages related to emergency preparedness and COVID-19 information among 120 churches with an estimated 12,000 congregants.^50^

This type of approach was mimicked through the BEC that convened a group of trusted medical and public health professionals in Pittsburgh and Allegheny County, PA, to address misinformation and specific community concerns related to SARS-CoV-2 infection, COVID-19 disease, vaccine development, and safety and efficacy.^13^ This team of trusted community advocates also designed and promoted culturally relevant and responsive advertisements and social media marketing tools.

Other efforts to address challenges to vaccine uptake might consider the following: (1) addressing mistrust, (2) combating misinformation, and (3) improving access to COVID-19 information and vaccines.^51^ Addressing mistrust should also include strategies such as having culturally appropriate, locally trusted, and responsive medical and public health professionals describe the benefits and challenges to vaccines,^52^ including community leaders and community representatives sharing their own stories related to vaccines and their decision-making process.^17^ Finally, infusing institutional equity throughout the process, as demonstrated through the R4P framework,^12,53^ is critical to ensure that all processes are community centered, take into account the historical contexts, eliminate the structures and practices that continue to exclude full participation from black communities, and identify the unique needs of communities and individuals historically and contemporarily oppressed.

### Strengths

To our knowledge, this study is the first to evaluate factors associated with COVID-19 vaccine hesitancy and uptake among black and African American residents in Allegheny County, Pennsylvania. Given the legacies and contemporaries of structural racism in this geographic region, it is imperative that research efforts actively collaborate with trusted community organizations and leaders; this study's partnership with the BEC and external, community-engaged recruitment methods intentionally cultivated relationships and built trust.

### Limitations

This study was limited by its recruitment methods, sample size, and cross-sectional assessment of influences to COVID-19 vaccine uptake. Several recruitment events were COVID-19 vaccination sites; therefore, the sample is likely not representative of the general black and African American population of the County. Social desirability bias may have influenced responses, such that participants did not want to report risky COVID-19 behaviors to public health practitioners on-site. Finally, questionnaires were conducted cross-sectionally, and so, it is impossible to assess how attitudes toward COVID-19 vaccinations have shifted as the pandemic continues into its third year.

## Conclusions

This study assessed factors related to COVID-19 vaccination among a sample of black adults from one geographic region in PA. While the majority of participants reported being fully vaccinated at the time of inquiry, addressing concerns among unvaccinated individuals in this region, including (1) transparency on contraindications and latent effects of vaccines, (2) clarity in the development and approval process for vaccines, (3) and receiving information from trusted individuals may allow for better discussion of the utility of vaccination. Future vaccine efforts should address mistrust, and promote transparency and ease and accessibility for better uptake.

## Supplementary Material

Supplemental data

## Supplementary Material

Supplementary Appendix SA1

## Abbreviations Used

aOR: adjusted odds ratio
BEC: Black Equity Coalition
CI: confidence interval
COVID-19: coronavirus disease 2019
FDA: Food and Drug Administration
OR: odds ratio
SD: standard deviation
