# Supplementary material for: Correlates of COVID-19 Vaccine Uptake in Black Adults Residing in Allegheny County, PA
Source: Health Equity. 2023 Aug 23;7(1):419–29. doi: 10.1089/heq.2022.0215 (PMC10457607; doi:10.1089/heq.2022.0215)
Supplement: Supplemental data [file Suppl_AppendixSA1.pdf]

**Vaccine Hesitancy Survey**

You are participating in research being conducted at the University of Pittsburgh. Our organization is partnering with the Black Equity Coalition (BEC) to gather residents' input on important questions related to access to healthcare, vaccination concerns and clinics, transportation, and issues we all are facing since the pandemic impacted our jobs and families.

BEC grew from a group of concerned professionals and community members of multiple races who came together in April 2020 to discuss the higher rates of COVID-19 deaths among the Black people in Allegheny County and nationally. Since our start we have increased access to information and vaccines for thousands (regardless of race) for those who want it.

Please take a few minutes to complete this survey to advocate for equitable healthcare for all. Participants will receive $15 as compensation for survey completion. There is a rare risk of breach of confidentiality that could arise from your participation. This is an anonymous questionnaire, and your responses will not be identifiable in any way.

Introductory eligibility questions:

Please say hello (*hidden field for bot detection)

- Please complete the following 3 questions to determine eligibility
- Are you 18 or older?
  - Yes
  - No (if no, survey concludes)
- Do you live in Allegheny County, PA?
  - Yes
  - No (if no, survey concludes)
- Do you identify as Black/African American?
  - Yes
  - No (if no, survey concludes)

- Demographics

Please tell us about yourself.

- What is your race (select all that apply)
    - White
    - Black or African American
    - American Indian or Alaska Native
    - Asian
    - Native Hawaiian
    - Other Pacific Islander
    - Other (Please specify)
- Are you Hispanic, Latino, or Spanish origin?
    - Yes
    - No
- What is your biological sex assigned at birth?
    - Male
    - Female
    - Intersex (individual born with non-binary sex characteristics)
    - None of these describe me
    - Prefer not to answer
- What is your age? (Fill in the blank)
- What is your highest level of education?
    - Some High School
    - High School graduate or GED
    - Some college
    - Trade/technical/vocational school
    - Associate degree
    - Bachelor’s degree
    - Master’s degree
    - Professional Degree
    - Doctorate Degree
- What community in Allegheny County, PA do you live in? (Fill in the blank)
- What is your zip code? (Fill in the blank)

- Are you employed? (Check all that apply)
    - Working part time
    - Working full time
    - Unemployed or not working
    - Retired
    - Student
- What kind of medical health insurance do you have?
    - Private insurance
    - Medicaid
    - Medicare
    - Other government insurance
    - None
- Would you say that (your) health in general is excellent, very good, good, fair, or poor?
    - Excellent
    - Very good
    - Good
    - Fair
    - Poor
- What is your income? (Select one)
    - Less than $20,000
    - $20,000 to $34,999
    - $35,000 to $49,999
    - $50,000 to $74,999
    - $75,000 to $99,999
    - Over $100,000
- Does your job offer you sufficient financial security? Y/N
- Do you expect your job situation change in the next 6 months? Y/N
- Are you currently receiving any support services or have other sources of income? (Check all that apply):
        - None
        - WIC

        - Social Security
        - Retirement and Pension Funds
        - Property Income
        - Child Support/Alimony
        - Food Stamp
        - Disability
        - Housing
        - Supplemental Security Income (SSI)
        - Public Assistance Payments
        - Veterans Payments
        - Survivor Benefits
        - Other (please specify)

- Thoughts on COVID safety strategies

We would like to know your thoughts on Covid-19 safety strategies. Please review the following statements and select the response that best applies.

    - Are you currently fully vaccinated for COVID-19 (received 1 shot of J&J or 2 shots of Moderna or Pfizer)? Yes/NO
    - If no, do you plan to receive the COVID-19 vaccination?
        - (YES) I have received my first dose and plan to receive the second
        - (YES/NO) I have received my first dose, but do not plan to receive the second
        - (NO/YES) I have not received the vaccine yet, but I would like to.
        - (Maybe) I may, I am waiting to see.
        - (NO) I am not going to get the vaccine.

    - If yes (to both, are you vaccinated and yes, they plan to receive the vaccine) -
        - What were the main reasons you chose to get the vaccine? Select all that apply.
            - My doctor/health provider(s) recommended the vaccine.
            - My job encouraged or required the vaccine.

                - I think vaccination is the best way to keep me from getting COVID-19 infection.
                - Getting the vaccine will help get life back to the way it was before the pandemic.
                - Getting the vaccine is a way that I can help end the COVID-19 pandemic.
                - I have a health condition that puts me at risk for severe COVID-19 infection.
                - A family member encouraged or made me get the vaccine.
                - So that I can spend time with friends and/or other family members again.
                - So that I can travel again.
                - The CDC guidelines recommend the vaccine.
                - Other (please specify)

            - Tell us more about the reasons you chose to get the vaccine.
            - Did/do you have any concerns before you got the vaccine?
                - (1) No Concerns
                - (2) A Few Concerns
                - (3) A Lot of Concerns
        - If you do not intend to get the vaccine or responded maybe –
            - What are the reasons for your choice? (Select all that apply)
                - I'm waiting to see
                - I need more information
                - I'm scared to get sick/of experiencing side effects
                - I don't know the long-term effects
                - I think the vaccine could give me COVID
                - Other (please specify)
- Intention to vaccinate children

If you are a parent or caregiver of a child under the age of 18, we would like to know your intention to vaccinate your child(ren). Please review the following statements and select the response that best applies.

- Are you the parent or guardian of any children aged 18 or younger?
    - Yes
    - No
        - (If yes #1) What are the ages of your child(ren) (check all that apply)
            - a. 12 and older
            - b. Younger than 12
- (If 12 and older) Has your child(ren) (aged 12 and older) received the vaccine for COVID-19?
    - Yes
    - No

- Do you plan to have your child(ren) (under age 12) receive the vaccine for COVID-19 when it is approved?
    - Yes
    - No
    - Not sure/maybe

        - If no, not sure, or maybe, why not?
            - I don't know where to get the vaccine for my child
            - I'm waiting to see
            - I need more information
            - I'm scared they will get sick/of experiencing side effects
            - I don't know the long-term effects
            - I think the vaccine could give them COVID-19
            - Other (please specify)

Please continue to the following questions to provide your thoughts on COVID-19 safety strategies.

- How effective do you feel masks are?

    - Extremely effective
    - Effective
    - Somewhat ineffective
    - Not effective at all
    - Not sure
- How often do you wear a mask?
    - All the time
    - Most of the time
    - Often
    - Sometimes
    - Rarely
    - Never
- How often do you wear a mask now compared to earlier in the pandemic?
    - More often
    - Less often
    - The same amount
- Which COVID safety measures have you taken during the pandemic? Select all that apply.
    - Social distancing
    - Maintained a social bubble
    - Wore a mask
    - Hand sanitizer or other sanitization methods
    - Delivered food or groceries
    - Gathered outdoors
    - Picked up food and ate at home
    - Worked remotely
    - Participated in virtual meetings, ceremonies, religious celebrations, and/or gatherings
    - Other:
    - None of these
- Are you able to practice these COVID safety measures at work?

        - All the time
        - Most of the time
        - Often
        - Sometimes
        - Rarely
        - Never
        - N/A
        - I don't know
- Are there any barriers to getting what you need during the pandemic?
        - Yes
            - If yes, please specify
        - No
- Have you tested positive for COVID?
        - Yes
            - If yes, how have your health needs changed since being infected by the coronavirus?
        - No
- Your input on vaccine clinics

We would like your input on COVID-19 vaccination opportunities in clinics near you. Please review the following statements and select the response that best applies.

    - Where should the County Health Department prioritize placing the next vaccination clinics?
        - Central
        - East End
        - North Side
        - South Side
        - West Side
        - Outside of the city
    - Specifically, in which neighborhood or municipality within the area you selected above?
    - Is there a health care center in your neighborhood or nearby?

        - Yes
        - No
    - If the vaccination was being administered at your neighborhood health care center, would you go there for the vaccine?
        - Yes
        - No
        - Not applicable – No clinic in my neighborhood
        - Not applicable – I already received the vaccine
    - What type of location would you be willing to go to in order to get a vaccine? (e.g., library, community center, church, etc.)
- Thoughts on general trust or mistrust of COVID-19 and the vaccine

We would like to know your thoughts on general trust and mistrust of COVID-19 and the vaccine. Please review the following statements and select the response that best describes how you feel to the best of your ability.

- A lot of information about COVID-19 is being held back by the government
    - Strongly agree
    - Agree
    - Neither agree nor disagree
    - disagree
    - Strongly disagree
- The government cannot be trusted to tell the truth about COVID-19
    - Strongly agree
    - Agree
    - Neither agree nor disagree
    - disagree
    - Strongly disagree
- The government is hiding information about COVID-19
    - Strongly agree
    - Agree
    - Neither agree nor disagree
    - disagree

        - Strongly disagree
    - Black people should be suspicious of information from the government about COVID-19
        - Strongly agree
        - Agree
        - Neither agree nor disagree
        - disagree
        - Strongly disagree
    - When it comes to COVID-19, the government is lying to us
        - Strongly agree
        - Agree
        - Neither agree nor disagree
        - disagree
        - Strongly disagree
    - COVID-19 is manmade
        - Strongly agree
        - Agree
        - Neither agree nor disagree
        - disagree
        - Strongly disagree
    - There is a cure for COVID-19, but it is being withheld from Black people
        - Strongly agree
        - Agree
        - Neither agree nor disagree
        - disagree
        - Strongly disagree
    - When it comes to COVID-19, Black people cannot trust health care providers
        - Strongly agree
        - Agree
        - Neither agree nor disagree
        - disagree

        - Strongly disagree
    - When it comes to COVID-19, doctors have the best interests of patients in mind
        - Strongly agree
        - Agree
        - Neither agree nor disagree
        - disagree
        - Strongly disagree
    - When it comes to COVID-19, Black people will receive the same medical care from health care providers as other groups
        - Strongly agree
        - Agree
        - Neither agree nor disagree
        - disagree
        - Strongly disagree

    - I have more trust in the Biden administration compared to the Trump administration when it comes to controlling the spread and treatment of COVID.
        - Strongly agree
        - Agree
        - Neither agree nor disagree
        - disagree
        - Strongly disagree
- Your doctor

We would like some information about your Doctor. Please review the following statements and select the response that best applies.

    - Do you currently have a primary care physician?
        - Yes
        - No
    - We are not asking you to move to a different doctor or change doctors. But the following question would apply if there may be times that you need to make an

appointment at a different doctor's office. Would you potentially use your neighborhood health care center for services?

        - (YES) I already use it for care.
        - (YES) I have not yet, but I would if needed.
        - (Maybe) I have not. But I might in the future.
        - (NO) I am not going to get care there.
        - (NO) There is no center in my neighborhood.

- Needs and Resources

Your needs and resources for support

    - Things are rough for many people at this time. What are some of the areas you feel are issues in your life or family?
    - Are you facing challenges in any of these areas?
        - Economic instability
            - Yes
            - No
        - Education access and quality
            - Yes
            - No
        - Healthcare access and quality
            - Yes
            - No
        - Neighborhood Issues
            - Yes
            - No
        - Issues in my community (social and community)
            - Yes
            - No
        - Other (Please specify)
    - Do you have reliable transportation to or for any of the following? (Check all that apply)
        - Medical care

        - Grocery stores
        - Other basic needs
- Social Media Use

We would like to know more about your social media use. Please review the following statements and select the response that best applies.

    - Select the social media platforms you use. Select all that apply.
        - Facebook
        - Instagram
        - Twitter
        - TikTok
        - Clubhouse
        - Reddit
        - YouTube
        - LinkedIn
        - Other (please specify)
        - None at all
    - Have you sought out information on COVID-19 vaccination on social media?
        - Yes/No
        - If yes –
            - Which platform are you most likely to use if/when looking for information on COVID-19 vaccination? Choose one.
                - Facebook
                - Instagram
                - Twitter
                - TikTok
                - Clubhouse
                - Reddit
                - YouTube
                - LinkedIn
                - Other (please specify)
                - None at all

            - When looking for information on vaccinations on social media, what format are you most likely to view?
                - Informative posts
                - Quick videos
                - Funny videos
                - Live events (Facebook live or IG)
                - Other (please specify)
        - If no –
            - Why have you not used social media to gather information about COVID?
                - Unreliable.
                - Don't use social media at all.
                - Prefer to get info from another source (list preferred source)

- Smoking Habits

Please tell us a little bit about your smoking habits.

- How often do you use tobacco and nicotine?
    - 1x daily
    - 3-5x daily
    - 6-10x daily
    - I use all day long to get through the day
    - I do not use tobacco
- Do you need support to quit using tobacco and nicotine products?
    - Yes
    - No
    - Maybe
- Have you ever attempted to quit smoking before?
    - Yes
    - No
- Do you currently use a vaping device or electronic cigarette/ e-cigarette as a smoking replacement?

    - Yes
    - No
    - Sometimes
- If you are a non-smoker, does someone in your home smoke?
    - Yes
    - No
- Do you agree that tobacco and nicotine products are harmful to your body?
    - Strongly agree
    - Agree
    - Neither agree nor disagree
    - Disagree
    - Strongly disagree
- Do you have access to tobacco and nicotine quit services in your neighborhood?
    - Yes
    - No
    - I do not know
